# Downstream signalling and specific inhibition of c-MET/HGF pathway in small cell lung cancer: implications for tumour invasion

**DOI:** 10.1038/sj.bjc.6603884

**Published:** 2007-07-31

**Authors:** P C Ma, M S Tretiakova, V Nallasura, R Jagadeeswaran, A N Husain, R Salgia

**Affiliations:** 1 Division of Hematology/Oncology, Department of Medicine, University Hospitals of Case Medical Center and Ireland Cancer Center, Case Western Reserve University, Case Comprehensive Cancer Center, Cleveland, OH 44106, USA; 2 Department of Pathology, University of Chicago Pritzker School of Medicine, and University of Chicago Cancer Research Center, Chicago, IL 60637, USA; 3 Section of Hematology/Oncology, Department of Medicine, University of Chicago Pritzker School of Medicine, and University of Chicago Cancer Research Center, Chicago, IL 60637, USA

**Keywords:** c-MET, signal transduction, small cell lung cancer, invasion

## Abstract

The c-MET receptor can be overexpressed, amplified, or mutated in solid tumours including small cell lung cancer (SCLC). In c-MET-overexpressing SCLC cell line NCI-H69, hepatocyte growth factor (HGF) dramatically induced c-MET phosphorylation at phosphoepitopes pY1230/1234/1235 (catalytic tyrosine kinase), pY1003 (juxtamembrane), and also of paxillin at pY31 (CRKL-binding site). We utilised a global proteomics phosphoantibody array approach to identify further c-MET/HGF signal transduction intermediates in SCLC. Strong HGF induction of specific phosphorylation sites in phosphoproteins involved in c-MET/HGF signal transduction was detected, namely adducin-*α* [S724], adducin-*γ* [S662], CREB [S133], ERK1 [T185/Y187], ERK1/2 [T202/Y204], ERK2 [T185/Y187], MAPKK (MEK) 1/2 [S221/S225], MAPKK (MEK) 3/6 [S189/S207], RB [S612], RB1 [S780], JNK [T183/Y185], STAT3 [S727], focal adhesion kinase (FAK) [Y576/S722/S910], p38*α*-MAPK [T180/Y182], and AKT1[S473] and [T308]. Conversely, inhibition of phosphorylation by HGF in protein kinase C (PKC), protein kinase R (PKR), and also CDK1 was identified. Phosphoantibody-based immunohistochemical analysis of SCLC tumour tissue and microarray established the role of c-MET in SCLC biology. This supports a role of c-MET activation in tumour invasive front in the tumour progression and invasion involving FAK and AKT downstream. The c-MET serves as an attractive therapeutic target in SCLC, as shown through small interfering RNA (siRNA) and selective prototype c-MET inhibitor SU11274, inhibiting the phosphorylation of c-MET itself and its downstream molecules such as AKT, S6 kinase, and ERK1/2. Investigation of mechanisms of invasion and, ultimately, metastasis in SCLC would be very useful with these signal transduction molecules.

The c-MET receptor tyrosine kinase (c-MET RTK) is the receptor for hepatocyte growth factor/scatter factor (HGF/SF; Jeffers *et al*, 1996). The mature HGF protein binds to its high-affinity receptor c-MET, leading to its activation and phosphorylation of multiple serine and tyrosine residue sites. The extracellular Sema domain of c-MET mediates binding to the ligand HGF, which, subsequently, leads to receptor dimerisation and activation of its intrinsic tyrosine kinase (Kong-Beltran *et al*, 2004; Stamos *et al*, 2004). The c-MET/HGF pathway has gained considerable interest through its apparent deregulation by overexpression or gain-of-function mutations in c-MET in various cancers, including lung cancer (Maulik *et al*, 2002c; Ma *et al*, 2003b; Christensen *et al*, 2005). We have shown that cell motility of small cell lung cancer (SCLC) cells is increased after HGF stimulation (Maulik *et al*, 2002a). Through PI3K pathway, HGF also stimulates activation of the cytoskeletal focal adhesion proteins paxillin, focal adhesion kinase (FAK) and PYK2 in SCLC (Maulik *et al*, 2002a, 2002b).

Small cell lung cancer is a very difficult disease with very poor prognosis and distant metastasis is often present at diagnosis (Murray *et al*, 2004). Small cell lung cancer comprises of about 15% of all lung cancers and is invariably associated with cigarette smoking. Novel therapy for this aggressive disease is urgently needed (Ma and Salgia, 2004; Sattler *et al*, 2004; Choong *et al*, 2005). The aggressiveness of the tumour is shown by its high propensity of organ invasion and metastasis to the brain, lymph nodes, liver, bone, leptomeninges, and also the bone marrow. Deregulation of cell motility may be tightly linked to tumour invasion, a process distinct from tumour progression. During invasion, cells degrade or remodel the surrounding extracellular matrix and migrate through the tissue boundary (Kermorgant *et al*, 2001). We have previously shown that c-MET/HGF pathway is functional and c-MET is often mutated in SCLC (Ma *et al*, 2003a). Our recent studies also show that c-MET is mutated in non-SCLC (NSCLC; Ma *et al*, 2005) and mesothelioma (Jagadeeswaran *et al*, 2006). Further studies of the role of c-MET/HGF signalling in SCLC will help to improve the understanding of the mechanism of invasion and metastasis in this aggressive disease.

The molecular mechanisms behind HGF-dependent invasive growth are not fully understood and have just begun to be elucidated. It has been suggested that c-MET leads to the induction of genes that are actively involved in invasion and metastasis. *In vivo*, the invasive-growth programming from c-MET/HGF signalling is thought to be an integrated function of a variety of biological responses such as cell proliferation and survival, cell dissociation/scattering, motility, induction of cell polarity, angiogenesis, wound healing, tissue regeneration, invasion, and tumour metastasis (Stella and Comoglio, 1999; Comoglio, 2001; Comoglio and Trusolino, 2002; Maulik *et al*, 2002c; Ma *et al*, 2003b).

Here we utilised a phosphoantibody array-based approach to study the phosphoproteome of SCLC c-MET/HGF signalling pathway. We have identified induction and inhibition of phosphorylation in numerous phosphoepitopes of phosphoproteins. These signalling pathway intermediates are found in diverse cellular regulatory signalling axis, including cell proliferation, survival, cell cycle, cytoskeletal functions, and transcription. With tumour tissue microarray (TMA) and phosphoantibody immunostaining, we also gained further insight into the role of c-MET/HGF signalling in SCLC biology and tumour invasion. Finally, novel targeted therapeutics against c-MET in SCLC was validated by small interfering RNA (siRNA) and the c-MET inhibitor SU11274.

## MATERIALS AND METHODS

### Cell lines and cell culture

Small cell lung cancer cell line NCI-H69 was purchased from the American Type Culture Collection (Rockville, MD, USA) and maintained in RPMI-1640 (Cellgro, Herndon, VA, USA) supplemented with 10% (v/v) fetal calf serum (FCS), L-glutamate, sodium pyruvate, and HEPES buffer as described previously (Ma *et al*, 2003a). Cells were deprived of growth factors by incubation in starvation media RPMI-1640 containing 0.5% (w/v) BSA (Sigma, St Louis, MO, USA) for 18 h before stimulation experiment with HGF (40 ng ml^−1^ for 7.5 min).

### c-MET inhibitor

SU11274 was provided by Pfizer Inc. (R&D, La Jolla, CA, USA) and was used as described previously (Ma *et al*, 2005). Small cell lung cancer NCI-H69 cells were treated with and without SU11274 in the presence of HGF (40 ng ml^−1^, 7.5 min) stimulation.

### Phosphoantibody array (Kinetworks™)

Global proteomics phosphoantibody array-based approach to analyse the signal transduction pathways of c-MET/HGF axis in SCLC NCI-H69 cell line was performed utilising the Kinetworks Phospho-Site Screen (KPSS)-1.3 and 2.0 (Kinexus, Vancouver, British Columbia, Canada). A wide range of phosphorylation site-specific antibodies were used in a qualitative and quantitative fashion as a specific assay for regulation of diverse cell signalling pathways (http://www.kinexus.ca/KPSS.htm). Kinetworks Phospho-Site Screen 1.3 and KPSS-2.0 track 31 and 37 known phosphorylation sites, respectively in phosphoproteins with antibodies that recognise specific phosphorylated epitopes of the target proteins (Supplementary Tables S1 and S2). A total of 350 *μ*g of whole cell lysates from H69 cells with or without HGF stimulation was used for each KPSS phosphoantibody array screen, which is an antibody-based method (Kinetworks) that relies on sodium dodecyl sulphate (SDS)-polyacrylamide minigel electrophoresis and multilane immunoblotters to permit the specific and quantitative detection of numerous protein kinases (PK) or other signal transduction proteins simultaneously (Pelech *et al*, 2003). Each blot was scanned densitometrically for quantitation, with each blot having its own unique Kinexus scan identification number. The trace quantity of a band was measured by the area under its intensity profile curve (units=intensity × mm). The trace quantity of a band is represented as c.p.m. corrected to a scan time of 60 s. The c.p.m. was then normalised to correct for differences in protein amount.

### Transfection and small interfering RNA

Small cell lung cancer cell line NCI-H69 was used in transfection study with siRNA targeting against c-MET as described previously (Ma *et al*, 2005), according to the manufacturer's instructions (Dharmacon Inc., Layayette, CO, USA).

### Immunoblotting, tumour tissue microarray and immunohistochemistry

Cellular proteins were extracted from whole cells using lysis buffer as described previously (Ma *et al*, 2005). Immunoblotting was performed using the following antibodies: anti-total c-MET (C-12; Santo Cruz Biotechnology, Santa Cruz, CA, USA), p-MET [Y1230/1234/1235] (BioSource, Camarillo, CA, USA), p-AKT [S473] (Abcam, Cambridge, MA, USA), p-ERK1/2 [T202/Y204] (BioSource), p-S6 kinase [T389] (Cell Signaling, Beverly, MA, USA), and *β*-actin (Santa Cruz) as loading control. Lung tumour tissue samples were collected with informed consent and in accordance with Institutional Review Board (IRB) approval protocols at the University of Chicago. Tumour tissue microarray was built using the ATA-27 Arrayer (1-mm punch size) from Beecher Instruments Inc. (Sun Prairie, WI, USA). The tumour microarray consists of nine SCLC tumour samples, and, as controls, two lung adnocarcinoma specimens. Corresponding normal lung or adjacent normal tissues were included in the microarray as negative controls as well. Each specimen was included in duplicates in the array. Tumour tissue immunohistochemistry (IHC) staining was performed using standard techniques as described previously (Ma *et al*, 2005) with antibodies against the following proteins: HGF (Zymed-Invitrogen, Carlsbad, CA, USA), c-MET (3D4 monoclonal; Zymed-Invitrogen), p-MET [Y1003] or [Y1230/1234/1235], p-FAK [Y861] (polyclonal; BioSource), FAK (Santa Cruz), p-AKT [S473] (Abcam), phosphotyrosine (p-Tyr; 4G10; Upstate, Lake Placid, NY, USA), and Ki-67 (mouse monoclonal: clone Ki-S5, DAKO, Carpinteria, CA, USA). In addition, similar IHC was performed on an archival paraffin-embedded SCLC tumour sections for topographic analysis of the c-MET/HGF signalling pathway.

## RESULTS

### c-MET/HGF signalling in small cell lung cancer identified via phosphoantibody array-based phosphoproteomics approach

**Figure 1:**
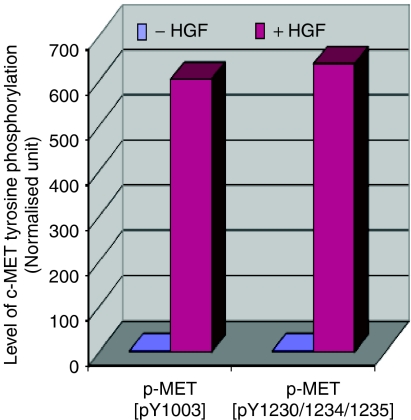
Induction of c-MET phosphorylation by HGF. HGF induction of tyrosine phosphorylation of c-MET at the phosphoepitopes p-MET [pY1003], the juxtamembrane c-CBL-binding site, and p-MET [pY1230/1234/1235], the cytoplasmic catalytic kinase domain autophosphorylation site as confirmed by immunoblotting is shown quantitatively here.

**Figure 2:**
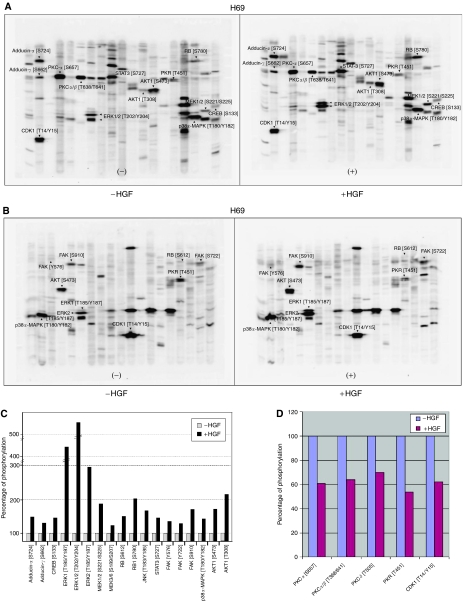
(**A**) KPSS-1.3 phosphoantibody multi-immunoblot array. SCLC NCI-H69 cells were treated with or without HGF (40 ng ml^−1^, 7.5 min). The WCLs were analysed using the KPSS-1.3 phosphoantibody multi-immunoblot array. The identities of phosphoprotein targets of interests are indicated by arrows and abbreviations. (**B**) KPSS-2.0 phosphoantibody immunoblot array. SCLC NCI-H69 cells were treated with or without HGF (40 ng ml^−1^, 7.5 min). The WCLs were analysed using the KPSS-2.0 phosphoantibody multi-immunoblot array The identities of phosphoprotein targets of interests are indicated by arrows and abbreviations. (**C**) c-MET/HGF signalling transduction in SCLC: phosphoproteins induced by HGF. The phosphoproteins downstream of c-MET found to be induced by HGF in SCLC in Kinetworks KPSS-1.3 and KPSS-2.0 are shown here. The specific phosphoepitopes induced are indicated. (**D**) c-MET/HGF signalling transduction in SCLC: phosphoproteins inhibited by HGF. The phosphoproteins downstream of c-MET found to be inhibited by HGF in SCLC in Kinetworks KPSS-1.3 and KPSS-2.0 are shown here. The specific phosphoepitopes inhibited are indicated.

We have previously demonstrated that c-MET/HGF signalling pathway is functional in SCLC NCI-H69 cell line (Maulik *et al*, 2002a, 2002b). The c-MET receptor tyrosine kinase is overexpressed in H69 cells and is inducible by exogenous HGF, resulting in induction of tyrosine phosphorylation at the major autophosphorylation sites pY1230/1234/1235 in the catalytic kinase domain, and also the pY1003 site in the juxtamembrane domain of c-MET (Figure 1). In addition, HGF induction of the c-MET receptor causes stimulation of cell motility and cell–cell aggregation of NCI-H69 cells in culture, correlating with induction of tyrosine phosphorylation of a number of focal adhesion proteins such as paxillin, FAK, and PYK2 (Maulik *et al*, 2002a). In our study, strong HGF induction of phosphorylation was readily detectable in a number of specific phosphorylation sites in phosphoproteins, downstream of c-MET itself, that are involved in c-MET/HGF signal transduction in SCLC NCI-H69 cells (Figures 2A and B).

A diverse set of phosphoproteins pivotal in a wide range of cellular regulation, consistent with the known pleiotropic effects of c-MET/HGF signalling, were identified. These include phosphoproteins that regulate transcriptional control: STAT3 [S727] and CREB [S133]; cell cycle G_1_/S checkpoint: RB [S612], RB1 [S780]; cell survival and apoptosis: AKT1 [S473] and [T308], JNK [T183/Y185]; cell proliferation and differentiation: MAPKK (MEK) 1/2 [S221/S225], ERK1 [T185/Y187], ERK2 [T185/Y187], ERK1/2 [T202/Y204]; stress and inflammatory response to cytokines and growth factors: MAPKK (MEK) 3/6 [S189/S207], p38*α*-MAPK [T180/Y182]; and also JNK [T183/Y185]; cytoskeletal functions: FAK [Y576/S722/S910], adducin-*α* [S724], and adducin-*γ* [S662]. Increased adducin expression has also been implicated in cell proliferation (Bowen *et al*, 2003).

Conversely, we also identified modest inhibition of phosphorylation by HGF in the following phosphoproteins (Figures 2A and B): PKC*α* [S657], PKC*α*/*β* [T368/641], and PKC*δ* [T505]. Moreover, HGF also inhibited phosphorylation of PKR [T451], which is known to have antiproliferative and pro-apoptotic functions. Lastly, HGF also reduced the threonine and tyrosine phosphorylation of the cell cycle checkpoint regulator CDK1 [T14/Y15].

### Downstream cellular signal transduction pathways induced by HGF

Compared with the untreated control of the SCLC NCI-H69 cells, HGF stimulation at 40 ng ml^−1^ for 7.5 min caused an induction of phosphorylation of the following phosphoprotein phosphosites (Figure 2C): adducin-*α* [S724] (146%), adducin-*γ* [S662] (125%), CREB [S133] (143%), ERK1 [T185/Y187] (449%), ERK1/2 [T202/Y204] (558%), ERK2 [T185/Y187] (289%), MAPKK (MEK) 1/2 [S221/S225] (183%), MAPKK (MEK) 3/6 [S189/S207] (118%), RB [S612] (146%), RB1 [S780] (197%), JNK [T183/Y185] (163%), STAT3 [S727] 139%), FAK [Y576] (132%), FAK [S722] (123%), FAK [S910] (165%), p38*α*-MAPK [T180/Y182] (136%), and AKT1 [S473] (166%) and [T308] (207%).

### Downstream cellular signal transduction pathways inhibited by HGF

Treatment of the H69 cells by HGF (40 ng ml^−1^; 7.5 min) caused a reduction of phosphorylation in the following phosphoproteins at the specified phosphosites (Figure 2D): PKC*α* [S657] (39% reduction), PKC*α*/*β* [T368/641] (36% reduction), PKC*δ* [T505] (30% reduction), PKR [T451] (46% reduction), and also CDK1 [T14/Y15] (38% reduction).

### c-MET/HGF signalling pathways in SCLC cytoskeletal functions

**Figure 3:**
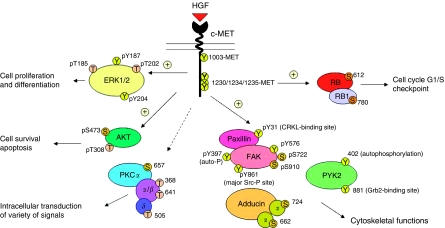
c-MET/HGF signal transduction pathways in SCLC. A schematic diagram to illustrate the versatile signalling functions of c-MET/HGF pathway in SCLC regulating various biological functions of the cells, including cytoskeletal functions, cell proliferation and differentiation, survival, and apoptosis is shown. Various potential serine, threonine and tyrosine phosphorylation sites on the signalling phosphoprotein intermediates are included. ⊕=stimulatory; ⊖=inhibitory.

Substantial evidence has been culminated to support the key role of c-MET/HGF signalling in mediating cell motility and cytoskeletal functions in SCLC (Maulik *et al*, 2002a, 2002b; Ma *et al*, 2003a). Phosphorylation of the focal adhesion proteins paxillin, FAK, and PYK2 are all inducible in response to HGF stimulation (Maulik *et al*, 2002a). Here, we also showed that HGF induced other phosphorylation sites on FAK, namely [Y576] (132%), [S722] (123%), and [S910] (165%; Figure 3). There have been reports on the role of PKC in focal adhesions and cell motility (Anilkumar *et al*, 2003). There are a number of phosphorylation sites on various PKC isoforms that can be inhibited by HGF, particularly PKC*α* [S657] (39% reduction), PKC*α*/*β* [T368/641] (36% reduction), and PKC*δ* [T505] (30% reduction). In SCLC NCI-H69 cells, HGF also induced phosphorylation on adducin-*α* [S724] (146%), and adducin-*γ* [S662] (125%), which have not been reported earlier.

### SCLC invasion as related to c-MET/HGF axis

**Figure 4:**
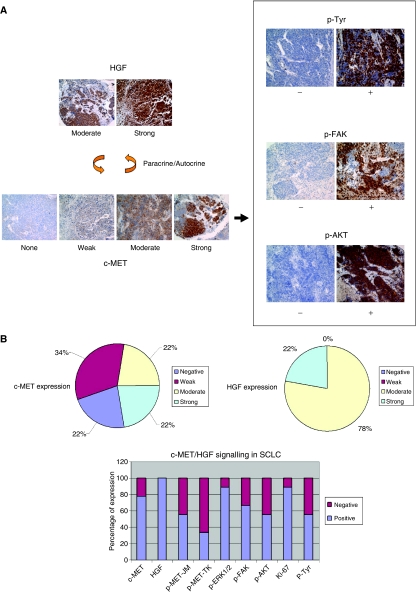
Tumour tissue microarray analysis of c-MET/HGF signalling. (**A**) Examples of the SCLC TMA tissues immunostained with HGF and c-MET receptor are shown. Also shown here in (**A**) are the phosphospecific immunostaining of their downstream signalling phosphoproteins in SCLC. Immunostain intensity: 0 (negative), 1+ (weak), 2+ (moderate), and 3+ (strong). Paracrine or autocrine signalling of c-MET/HGF axis leads to downstream signalling activation and is shown here with examples of immunostaining using p-Tyr, p-FAK and p-AKT, (−) negative, (+) positive. (**B**) Quantitative expression of the c-MET/HGF axis and its downstream signalling phosphoproteins in SCLC.

To understand better the role of the *in vivo* c-MET/HGF signalling in SCLC tumour tissues, we performed IHC analysis in SCLC tumours, as established on a tissue microarray. Various phosphospecific antibodies were used in the IHC analysis to provide both qualitative and quantitative information of the signalling pathways in the tumours (Figure 4). We found that there was 100% positive (moderate, 78% (7/9); strong, 22% (2/9)) expression of HGF in SCLC, with predominantly intratumoural cytoplasmic staining pattern. This finding supports the notion of an autocrine c-MET/HGF signalling in SCLC. There was 78% (7/9) of SCLC expressing c-MET positively, in which 42% (3/7) had weak, 29% (2/7) had moderate, and 29% (2/7) had strong expression. Furthermore, we identified 56% (5/9) pY1003-MET and 33% (3/9) pY1230/1234/1235-MET-positive expression in the SCLC TMA.

**Figure 5:**
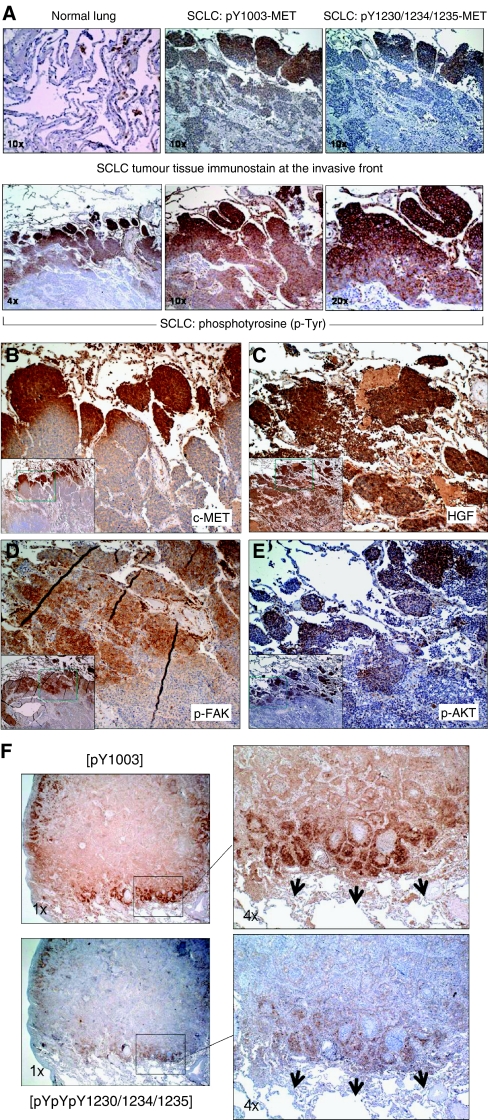
Topographic analysis of the invasive front of SCLC using phosphoantibody IHC. (**A**) Topographic role of p-MET and phosphoproteins with pTyr activation. (**B**) Overexpression of c-MET along the SCLC invasive tumour front, × 10. Inset: × 4. (**C**) Overexpression of HGF in SCLC tumour tissue, × 10. Inset: × 4. (**D**) Topographic role of activated cytoskeletal focal adhesion protein p-FAK, × 10. Inset: × 4. (**E**) Topographic role of activated survival signalling molecule p-AKT, × 10. Inset: × 4. (**F**) Preferential expression of activated p-MET along the tumour invasive front in lung adenocarcinoma. Upper panel, p-MET [Y1003]. Lower panel, p-MET [pY1230/1234/1235]. Magnification: left, × 1; right, × 4.

There were 56% (5/9) SCLC samples that had p-Tyr expression, all with strong (3+) IHC staining. It is interesting to note that p-ERK1/2 staining was uniformly strong (3+) in its staining pattern in 89% (8/9) positive samples. The Ki-67 staining was positive in 89% (8/9) SCLC samples. Positive staining in p-FAK [pY861] and p-AKT [pS473] were seen in 67 and 56% of samples, respectively (Figure 5).

Tumour tissue microarray analysis allows simultaneous analysis of a number of different phosphoproteins both within the same tumour tissue core and also among different tumour tissues. Analysis of both the IHC staining patterns of p-MET and Ki-67 indicates that the strong staining intensities do not coincide within the same tumours, suggesting that activated p-MET does not necessarily activate the cell proliferation pathway. On the other hand, p-MET (especially pY1003) staining coincided with p-FAK and p-AKT expression, suggesting the role of c-MET activation in cell migration, invasion, and survival. None of the three normal lung or paired normal tissues in the TMA showed any expression of either c-MET or p-MET.

### Analysis of c-MET/HGF signalling activation in SCLC tumour tissues

We also studied the role of c-MET/HGF signalling pathway in SCLC tumour tissues using phosphospecific antibodies IHC analysis with focus on its topographic pattern of expression. The downstream signalling molecules p-AKT [S473] and p-FAK [Y861] were studied in addition to HGF, c-MET, p-MET (both Y1003 and Y1230/1234/1235), and p-Tyr. In one of the four SCLC tumour tissues (25%) screened, preferential c-MET overexpression and activation of p-MET (both the phosphoepitopes pY1003 and pY1230/1234/1235) along the tumour expanding invasive front were identified (Figures 5A and B). Similar observation was also made in NSCLC tumour specimens (Figure 5F; Ma *et al*, 2005). Hepatocyte growth factor staining was more uniform within the SCLC tumour, with only slightly stronger staining along the invasive edge (Figure 5C). Moreover, preferential staining with p-FAK [Y861], p-AKT [S473], and also p-Tyr antibody (similar to that of c-MET and p-MET) was seen along the invasive front in SCLC (Figures 5D and E). Particularly evident in the case of anti-p-Tyr immunostaining, there was an outwardly increasing gradient of IHC staining intensity along the axis from the core towards the peripheral invasive front (Figure 5A). The other three SCLC tumour tissues screened were immunostained negative for both of the p-MET antibodies.

### Activated p-MET as a potential target for therapeutic inhibition

#### Validation by siRNA against c-MET

**Figure 6:**
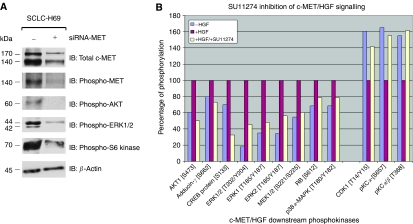
c-MET as potential target for therapeutic inhibition – siRNA validation (**A**) and c-MET inhibitor SU11274 (**B**). (**A**) c-MET-specific siRNA (+) was used to downregulate c-MET expression in SCLC NCI-H69 cells in growth media containing 10% FCS as described previously (Ma *et al*, 2003a, 2005). Cells treated with mock siRNA scramble sequence were included as negative control (−). The WCLs were collected 72 h after siRNA transfection and analysed on 7.5% SDS–PAGE, and electrotransferred onto Immobilon-P membrane, and immunoblotted using the following antibodies: anti-total c-MET (top panel), p-MET [pY1230/1234/1235] (second panel), p-AKT [S473] (third panel), p-ERK1/2 [T202/Y204] (fourth panel), p-S6 kinase [T389] (fifth panel), and *β*-actin (bottom panel, loading control). (**B**) The WCLs of H69 cells treated with or without HGF and with or without SU11274 were collected and analysed on the phosphoantibody-based multilane immunoblotting and quantitated as described in Materials and Methods. A number of phosphoproteins were shown to be modulated by SU11274, both in the positive and negative fashion, in its inhibition against the c-MET/HGF signalling.

Next, we investigated the potential role of targeting c-MET to inhibit SCLC. We utilised c-MET-specific siRNA to knock down the c-MET signalling in the SCLC NCI-H69 cells using standardised techniques as described in the Materials and methods (Ma *et al*, 2005; Figure 6A). c-MET receptor was substantially downregulated by siRNA-MET which also correlated with a concomitant inhibition of p-MET as well as its downstream signalling molecules p-AKT, p-ERK1/2, and p-S6 kinase (Figure 6A).

#### SU11274 inhibition of c-MET/HGF signalling

We have previously characterised and described the efficacy of the specific small molecule inhibitor of c-MET (Sattler *et al*, 2003; Ma *et al*, 2005). Here, we tested the inhibitor against the SCLC NCI-H69 cells in the phosphokinase screen to study its effect on c-MET/HGF signalling pathway components (Figure 6B). The HGF-stimulated phosphorylation of the following downstream phosphokinases was inhibited by SU11274: p-ERK1 [T202/Y204], p-ERK1/2 [T185/Y187], p-MEK1/2 [S221/S225], p38*α* p-MAP kinase [T180/Y182], p-AKT1 [S473], p-RB [S672], p-adducin-*γ* [S662], and p-CREB [S133]). SU11274 was also effective in abrogating the inhibitory effect of HGF on the specific phosphorylation of p-PKC*α* [S657], p-PKC*α/β* [T368], and p-CDK1 [T14/Y15] (Figure 6B).

## DISCUSSION

The c-MET is a key receptor tyrosine kinase expressed predominantly in epithelial cells. The c-MET has been identified as an oncogene with convincing evidence, demonstrating the direct key roles of activating c-MET mutations and met amplification in promoting tumorigenesis *in vivo* (Graveel *et al*, 2004, 2005). We have previously demonstrated that c-MET/HGF pathway not only is functional in SCLC, it also harbours novel mutations of c-MET in the semaphorin and juxtamembrane domains (Maulik *et al*, 2002a; Kijima *et al*, 2003; Ma *et al*, 2003a). Here, we further investigated the c-MET/HGF signalling pathway in SCLC with focus on the phosphoproteome.

Hepatocyte growth factor dramatically induced phosphorylation of c-MET at its various serine threonine tyrosine epitopes, including the major autophosphorylation sites pY1230/1234/1235 within the catalytic tyrosine kinase domain, and the regulatory juxtamembrane c-CBL-binding site pY1003. Hepatocyte growth factor also dramatically enhanced SCLC cell motility with concomitant induction of tyrosine phosphorylation of a number of cellular proteins such as the focal adhesion proteins paxillin, focal adhesion kinase (FAK), and PYK2. Here, we adopted a global phosphoantibody array-based approach to delineate further the c-MET/HGF signal transduction pathway and its downstream signalling intermediates in the SCLC phosphoproteome. Using SCLC NCI-H69 cells with and without HGF stimulation as the model, the screening arrays KPSS-1.3 and KPSS-2.0 together allowed detection of strong HGF-induction of specific phosphorylation sites in phosphoproteins downstream of c-MET itself, that are involved in diverse cellular regulation, including transcriptional control, cell cycle G_1_/S checkpoint, cell survival and apoptosis, cell proliferation and differentiation, stress and inflammatory response to cytokines and growth factors, as well as cytoskeletal functions. Phosphoprotein epitopes that are inhibited by HGF in their phosphorylation were also identified.

We have previously shown that cell motility of SCLC is enhanced by ligand stimulation with HGF via c-MET RTK (Weisberg *et al*, 1997; Maulik *et al*, 2002a). The serum level of HGF is significantly higher in SCLC patients than that in normals. Moreover, serum HGF level higher than 500 pg ml^−1^ is associated with a trend towards worse survival (Bharti *et al*, 2004). The mechanism whereby HGF activation of c-MET leads to increased motility, migration, and invasion in cancer cells has not been well-defined. Hepatocyte growth factor-induced c-MET activation leads to increased membrane ruffling, filopodia formation, and also motility/migration in SCLC (Maulik *et al*, 2002a). Cell motility is also regulated by the focal adhesions. c-MET/HGF signalling has been shown to induce the formation of focal adhesions (Matsumoto *et al*, 1995). The focal adhesion is comprised of multiple nonenzymatic proteins including vinculin, *α*-actinin, paxillin, and kinases such as FAK and PYK2. Focal adhesion kinase is a 125 kDa protein, consisting of an N-terminal integrin-binding site, a central kinase domain, and a C-terminal focal adhesion-targeting and paxillin-binding domains (Schaller and Sasaki, 1997; Mitra *et al*, 2005). Focal adhesion kinase family members include proteins such as PYK2 and FAK-B. Focal adhesion kinase is important receptor-proximal regulator of cell shape, adhesion, and cell motility. Focal adhesion kinase was discovered as a substrate of SRC and is key to integrin signalling. It interacts with integrins and other focal adhesion proteins such as paxillin, regulatory enzymatic signalling molecules such as PI3K, SH3 domain-containing adapter proteins, and other tyrosine kinases via an autophosphorylation site at tyrosine residue 397 (pY397). Activation of the pY397 autophosphorylation site of FAK promotes SRC binding, leading to the conformational activation of SRC, and, subsequently, a dual activated FAK–SRC signalling complex (Mitra *et al*, 2005). Within this FAK–SRC complex, SRC phosphorylates FAK at pY861, associating with an increase in SH3-domain-mediated binding of p130Cas to the FAK C-terminal proline-rich regions, which, in turn, promotes cell motility and invasion (Lim *et al*, 2004). Focal adhesion kinase [pY861] is also crucial for RAS-mediated transformation of fibroblast (Lim *et al*, 2004). We have recently shown that FAK was phosphorylated on pY397 (autophosphorylation site) and pY861 (the major SRC phosphorylation site) in response to HGF in SCLC (Maulik *et al*, 2002a). With regard to cell motility and migration, overexpression of FAK in MDCK cells apparently enhances the cell migration component of the HGF-induced cell scattering. Here, we also identified p-FAK [Y576], [S722], and also [S910] to be induced by c-MET/HGF in H69 cells. Focal adhesion kinase plays a central role in cell spreading, differentiation, migration, cell death, and acceleration of the G_1_ to S phase transition of the cell cycle. Tyr576 and Tyr577 are located in the kinase activation loop of FAK and, when phosphorylated by SRC, results in maximal activity. The role of phosphorylation at Ser722 and Ser910 is currently being actively investigated. While SRC is a known intermediate in c-MET/HGF signalling, we did not observe induction of p-SRC [Y418] or [Y529] as included in the KPSS screens. These specific phosphoepitope sites might not be involved in the SRC activation by HGF-stimulated c-MET signalling. Further work to catalogue various specific phosphoepitope induction in the downstream signalling intermediates of c-MET/HGF pathway through global phosphoproteomics analysis would be very useful.

Cellular molecules regulating tumour cell motility and migration are believed to be key element in promoting tumour invasion. Evidence of signalling pathways regulating tumour cell invasion may be found within the tumour itself through detailed IHC analysis. The use of TMA can provide a platform to study a number of different signalling molecules simultaneously on the multiple tumour specimens, allowing both quantitative and qualitative analyses. Evidence to support the autocrine and paracrine regulation of c-MET pathway was provided in the TMA analysis in this study. Hepatocyte growth factor was immunostained extensively in 100% of all the tumour tissues examined, and there was intratumoural staining as well. Furthermore, its staining is more uniform across the tumour tissue itself without preferential overexpression topographically. Interestingly, there is a discordant staining pattern between p-MET and Ki-67 in the TMA, suggesting that activation of p-MET might not always be responsible for the SCLC cell proliferation and other regulatory pathways might be at play. On the other hand, the immunostaining of p-FAK [Y861] and p-AKT [S473] correlated well with that of the p-MET, suggesting that c-MET is upstream of the two signalling molecules FAK and AKT.

We have also examined in details the topographic distribution of the various phosphoproteins in the c-MET/HGF pathway in SCLC tumour tissue. Preferential staining of p-MET along the expanding invasive front of the SCLC and adenocarcinoma tumour (Figure 5; Ma *et al*, 2005) was evident. It suggests that there is preferential activation of the c-MET receptors along the tumour invasive front compared with the tumour core. Our findings here differ from the recent report of the induction of c-MET overexpression by hypoxia (Pennacchietti *et al*, 2003), a cellular state that is conceivably more prominent within the tumour core than along the peripheral expanding tumour front juxtaposing the adjacent lung alveoli. Here, c-MET is found to be preferentially overexpressed and activated along the peripheral tumour invasive front. Similarly, it is also true for p-MET, as well as p-FAK and p-AKT, again supporting the role of c-MET activation in cell survival, motility, invasion, and metastasis in SCLC. Moreover, there was a gradient of phosphotyrosine (p-Tyr) staining in the tumour tissue examined (increasing gradient outward to tumour periphery). This finding suggests that there are molecules in the tyrosine phosphoproteome, some of which are likely to be downstream of c-MET under its regulation, preferentially involved in the regulation of SCLC tumour invasion and metastasis along the invasive tumour front. In particular, FAK is an important tyrosine kinase in the control of cytoskeletal function and cell motility. FAK overexpression has also been shown to have synergistic effect with HGF on cell transformation (Chan *et al*, 2002). Focal adhesion kinase activation can also promote aggressive uveal and cutaneous melanoma phenotype (Hess *et al*, 2005).

Recent molecular targeting in SCLC using c-KIT inhibitor (Gleevec) and antisense BCL-2 (Oblisense) has been far from successful (Johnson *et al*, 2003; Rudin *et al*, 2004). While SCLC is mostly chemosensitive in frontline therapy, the inevitable disease relapse in most patients and the subsequent chemoresistance remain formidable problems leading to very poor overall outcome (Ma and Salgia, 2004). The c-MET would be an attractive therapeutic target to be inhibited in SCLC to expand the therapeutic armamentarium. We show here that siRNA inhibition of c-MET in SCLC significantly downregulates the activation of p-AKT and p-S6 kinase in the cell survival pathway, and p-ERK1/2 in the proliferation pathway. Furthermore, the c-MET inhibitor SU11274 can inhibit the activation of c-MET/HGF and its downstream signal transducers. These data support further studies and clinical development of inhibitors targeting c-MET in SCLC (Ma *et al*, 2003b, 2005; Sattler *et al*, 2003, 2004; Christensen *et al*, 2005). c-MET/HGF-targeted therapy such as the specific inhibitor SU11274 would be promising in at least a subset of SCLC patients with deregulated c-MET signalling through its overexpression or activating mutations. Multi-targeted kinase inhibitors to target c-MET together with VEGFR2 (XL880; Exelixis Inc., San Francisco, CA, USA) is also currently under development. Moreover, antibody approach to inhibit the c-MET/HGF pathway is also under investigation, both using antibodies against the ligand (Burgess *et al*, 2006) as well as the receptor (Nguyen *et al*, 2003). Development of techniques of tumour tissue IHC with phosphoantibodies of c-MET and its downstream signal transducers molecules would enable the validation of inhibitor efficacy in the tumour tissue itself in future clinical studies of c-MET inhibitors.
