# Supplementary material for: Downstream signalling and specific inhibition of c-MET/HGF pathway in small cell lung cancer: implications for tumour invasion
Source: Br J Cancer. 2007 Jul 31;97(3):368–77. doi: 10.1038/sj.bjc.6603884 (PMC2360323; doi:10.1038/sj.bjc.6603884)
Supplement: Supplementary Tables S1 and S2 [file 6603884x1.doc]

| | | **NAME OF PROTEIN** | **ABBREV** | **EPITOPE** | | --- | --- | --- | | **Adducin alpha** | **Adducin α** | **S724** | | **Adducin gamma** | **Adducin γ** | **S662** | | **cAMP Response Element Binding Protein** | **CREB** | **S133** | | **Cyclin-dependent kinase 1 (cdc2)** | **CDK1** | **Y15** | | **dsRNA dependent protein kinase** | **PKR** | **T451** | | **Extracellular regulated kinase 1/2** | **ERK1/2** | **T202/Y204** | | **Extracellular regulated kinase 2** | **ERK2** | **T185/Y187** | | **Glycogen synthase kinase 3 alpha** | **GSK3α** | **S21** | | **Glycogen synthase kinase 3 alpha** | **GSK3α** | **Y279** | | **Glycogen synthase kinase 3 beta** | **GSK3β** | **S9** | | **Glycogen synthase kinase 3 beta** | **GSK3β** | **Y216** | | **MAP kinase kinase 1/2** | **MEK1/2** | **S221/S225** | | **MAP kinase kinase 3/6** | **MEK3/6** | **S189/S207** | | **MAP kinase kinase 6 (MEK6)** | **MEK3/6** | **S207** | | **Mitogen-and stress-activated protein kinase 1/2** | **MSK1/2** | **S376** | | **Mitogen-and stress-activated protein kinase 1/2** | **MSK1/2** | **S376** | | **N-methyl-D-aspartate glutamate receptor subunit 1** | **NR1** | **S896** | | **Oncogene JUN** | **JUN** | **S73** | | **Oncogene Raf 1** | **RAF1** | **S259** | | **Oncogene SRC** | **SRC** | **Y529** | | **Oncogene SRC** | **SRC** | **Y418** | | **p38 alpha MAP kinase** | **p38 MAPK** | **T180/Y182** | | **Protein kinase B alpha (Akt1)** | **Akt1** | **S473** | | **Protein kinase B alpha (Akt1)** | **Akt1** | **T308** | | **Protein kinase C alpha** | **PKCα** | **S657** | | **Protein kinase C alpha/beta** | **PKCα/β** | **T638/641** | | **Protein kinase C delta** | **PKCδ** | **T505** | | **Protein kinase C epsilon** | **PKCε** | **S719** | | **Retinoblastoma 1** | **RB** | **S780** | | **Retinoblastoma 1** | **RB** | **S807/S811** | | **Ribosomal S6 kinase 1** | **RSK1** | **T360/S364** | | **S6 kinase p70** | **p70 S6K** | **T389** | | **Signal transducer and activator of transcription 1** | **STAT1** | **Y701** | | **Signal transducer and activator of transcription 3** | **STAT3** | **S727** | | **Signal transducer and activator of transcription 5** | **STAT5** | **Y694** | | **SMA-and MAD-related protein 1** | **SMAD1** | **S463/465** | | **Stress-activated protein kinase (JNK)** | **SAPK** | **T183/Y185** | | | --- | --- | --- | --- | --- | --- | --- | --- | --- | --- | --- | --- | --- | --- | --- | --- | --- | --- | --- | --- | --- | --- | --- | --- | --- | --- | --- | --- | --- | --- | --- | --- | --- | --- | --- | --- | --- | --- | --- | --- | --- | --- | --- | --- | --- | --- | --- | --- | --- | --- | --- | --- | --- | --- | --- | --- | --- | --- | --- | --- | --- | --- | --- | --- | --- | --- | --- | --- | --- | --- | --- | --- | --- | --- | --- | --- | --- | --- | --- | --- | --- | --- | --- | --- | --- | --- | --- | --- | --- | --- | --- | --- | --- | --- | --- | --- | --- | --- | --- | --- | --- | --- | --- | --- | --- | --- | --- | --- | --- | --- | --- | --- | --- | --- | --- | |
| --- | --- | --- | --- | --- | --- | --- | --- | --- | --- | --- | --- | --- | --- | --- | --- | --- | --- | --- | --- | --- | --- | --- | --- | --- | --- | --- | --- | --- | --- | --- | --- | --- | --- | --- | --- | --- | --- | --- | --- | --- | --- | --- | --- | --- | --- | --- | --- | --- | --- | --- | --- | --- | --- | --- | --- | --- | --- | --- | --- | --- | --- | --- | --- | --- | --- | --- | --- | --- | --- | --- | --- | --- | --- | --- | --- | --- | --- | --- | --- | --- | --- | --- | --- | --- | --- | --- | --- | --- | --- | --- | --- | --- | --- | --- | --- | --- | --- | --- | --- | --- | --- | --- | --- | --- | --- | --- | --- | --- | --- | --- | --- | --- | --- | --- | --- |

# Table S1. Kinetworks^TM^ Phospho-Site Screen 1.3 (KPSS-1.3) phosphoprotein list.

| | | **FULL NAME OF PROTEIN** | **ABBREV** | **EPITOPE** | | --- | --- | --- | | **Proto-oncogene tyrosine-protein kinase Kit** | **c-Kit** | **Y703** | | **c-Kit Receptor Tyrosine Kinase** | **c-Kit** | **Y730** | | **c-Kit Receptor Tyrosine Kinase** | **c-Kit** | **Y936** | | **c-MET Receptor Tyrosine Kinase** | **c-MET** | **Y1003** | | **c-MET Receptor Tyrosine Kinase** | **c-MET** | **Y1230/Y1234/Y1235** | | **Cyclin-dependent kinase 1** | **CDK1** | **T14/Y15** | | **Epidermal Growth Factor Receptor** | **EGFR** | **Y1068** | | **Epidermal Growth Factor Receptor** | **EGFR** | **Y1148** | | **ErbB2 receptor tyrosine kinase** | **ErbB2** | **Y1139** | | **Eukaryotic Translation Initiation Factor 2B epsilon subunit** | **eIF-2Be** | **S539** | | **Extracellular signal-regulated kinase 1** | **ERK1** | **T185/Y187** | | **Extracellular signal-regulated kinase 2** | **ERK2** | **T185/Y187** | | **Focal Adhesion Kinase** | **FAK** | **S722** | | **Focal Adhesion Kinase** | **FAK** | **S910** | | **Focal Adhesion Kinase** | **FAK** | **Y576** | | **Focal Adhesion Kinase** | **FAK** | **Y577** | | **Insulin Receptor** | **IR** | **Y972** | | **Insulin Receptor Substrate-1** | **IRS1** | **Y1179** | | **Insulin Receptor Substrate-1** | **IRS1** | **Y612** | | **Insulin/Insulin-Like Growth Factor-1 Receptor** | **IR/IGF1-R** | **Y1162/Y1163** | | **Lymphocyte-specific protein kinase** | **Lck** | **S158** | | **Lymphocyte-specific protein kinase** | **Lck** | **Y192** | | **Lymphocyte-specific protein kinase** | **Lck** | **Y505** | | **MAP Kinase Kinase 1** | **MEK1** | **S298** | | **MAP Kinase Kinase 1** | **MEK1** | **T292** | | **MAP Kinase Kinase 1** | **MEK1** | **T386** | | **MAP Kinase Kinase 2** | **MEK2** | **T394** | | **Oncogene SRC** | **SRC** | **Y418** | | **Oncogene SRC** | **SRC** | **Y529** | | **p38 alpha MAP kinase** | **p38 MAPK** | **T180/Y182** | | **Protein kinase B alpha (Akt1)** | **Akt1** | **S473** | | **Protein kinase R** | **PKR** | **T451** | | **Retinoblastoma protein** | **RB** | **S612** | | **SH2 domain-containing transfroming protein 1** | **Shc** | **Y239/Y240** | | **Stress-activated protein kinase (JNK)** | **SAPK (JNK)** | **T183/Y185** | | **Tumor suppressor p53** | **p53** | **S392** | | | --- | --- | --- | --- | --- | --- | --- | --- | --- | --- | --- | --- | --- | --- | --- | --- | --- | --- | --- | --- | --- | --- | --- | --- | --- | --- | --- | --- | --- | --- | --- | --- | --- | --- | --- | --- | --- | --- | --- | --- | --- | --- | --- | --- | --- | --- | --- | --- | --- | --- | --- | --- | --- | --- | --- | --- | --- | --- | --- | --- | --- | --- | --- | --- | --- | --- | --- | --- | --- | --- | --- | --- | --- | --- | --- | --- | --- | --- | --- | --- | --- | --- | --- | --- | --- | --- | --- | --- | --- | --- | --- | --- | --- | --- | --- | --- | --- | --- | --- | --- | --- | --- | --- | --- | --- | --- | --- | --- | --- | --- | --- | --- | |
| --- | --- | --- | --- | --- | --- | --- | --- | --- | --- | --- | --- | --- | --- | --- | --- | --- | --- | --- | --- | --- | --- | --- | --- | --- | --- | --- | --- | --- | --- | --- | --- | --- | --- | --- | --- | --- | --- | --- | --- | --- | --- | --- | --- | --- | --- | --- | --- | --- | --- | --- | --- | --- | --- | --- | --- | --- | --- | --- | --- | --- | --- | --- | --- | --- | --- | --- | --- | --- | --- | --- | --- | --- | --- | --- | --- | --- | --- | --- | --- | --- | --- | --- | --- | --- | --- | --- | --- | --- | --- | --- | --- | --- | --- | --- | --- | --- | --- | --- | --- | --- | --- | --- | --- | --- | --- | --- | --- | --- | --- | --- | --- | --- |

**Table S2. Kinetworks^TM^ Phospho-Site Screen 2.0 (KPSS-2.0) phosphoprotein list.**
